# Abundance and Diversity of Soil Arthropods in the Olive Grove Ecosystem

**DOI:** 10.1673/031.012.2001

**Published:** 2012-02-09

**Authors:** Maria Fátima Gonçalves, José Alberto Pereira

**Affiliations:** ^1^ CITAB, Centre for the Research and Technology of Agro-Environment and Biological Sciences, University of Trásos-Montes and Alto Douro, Apt. 1013, 5001-801, Vila Real, Portugal; ^2^ CIMO, School of Agriculture, Polytechnic Institute of Bragança, Campus Sta Apolónia, Apartado 1171, 5301-854 Bragança, Portugal

**Keywords:** day period, Formicidae, pitfall trap, soil fauna

## Abstract

Arthropods are part of important functional groups in soil food webs. Recognizing these arthropods and understanding their function in the ecosystem as well as when they are active is essential to understanding their roles. In the present work, the abundance and diversity of soil arthropods is examined in olive groves in the northeast region of Portugal during the spring. Five classes of arthropods were found: Chilopoda, Malacostraca, Entognatha, Insecta, and Arachnida. Captures were numerically dominated by Collembola within Entognatha, representing 70.9% of total captures. Arachnida and Insecta classes represented about 20.4 and 9.0%, respectively. Among the predatory arthropods, the most representative groups were Araneae and Opiliones from Arachnida, and Formicidae, Carabidae, and Staphylinidae from Insecta. From the Formicidae family, *Tetramorium semilaeve* (Andre 1883), *Tapinoma nigerrimum* (Nylander 1856), and *Crematogaster scutellaris* (Olivier 1792) were the most representative ant species. Arthropods demonstrated preference during the day, with 74% of the total individuals recovered in this period, although richness and similarity were analogous during the day and night.

## Introduction

The soil is an extremely dynamic, complex, and highly heterogeneous system that allows the development of an extremely large number of ecological habitats, is the home of an array of live organisms, and performs important functions for the ecosystem (Gardi and Jeffery 2009).

The most dominant groups of soil organisms are microorganisms, such as bacteria and fungi, followed by a huge variety of animals such as nematodes, arthropods, enchytraeids, and earthworms (Jeffery et al. 2010). In the soil, these organisms have central functions in organic matter decomposition, the nutrient cycle, the enhancement of soil structure, and the control of soil organisms, including crop pests (Moore and Walter 1988). However, soil organisms also contribute to the regulation of atmospheric composition and climate, water quantity and quality, and the reduction of environmental pollution (Gardi and Jeffery 2009; Jeffery et al. 2010; Lavelle et al. 2006). Furthermore, they are important components of soil food webs (Gardi and Jeffery 2009; Jeffery et al. 2010). According to these functions, the organisms and microorganisms that live in the soil have been divided into three wide functional groups, i.e., chemical engineers, biological regulators, and ecosystem engineers (Turbé et al. 2010; Lavelle et al. 2006).

Identifying patterns and determinants of species richness is vital and is of fundamental importance to the management and preservation of biological diversity (Bardgett 2002), and is strongly recommended for the integrated production of olives (Malavolta et al. 2002).

The present study reports the biodiversity of soil arthropods in olive groves from Terra Quente to help understand the role that they play in the soil. Particular emphasis was given to generalist predators that feed on olive enemies, such as the olive fruit fly, which spends part of its life cycle in the soil.

## Materials and Methods

### Experimental site

The study was conducted in three traditional groves in the Terra Quente region (Northeast of Portugal) near Mirandela (41° 30′ N, 7° 10′ W). The groves, hereafter designated as Paradela A, Paradela B, and Valbom dos Figos (V. Figos), were non-irrigated and were not submitted to phytossanitary treatments. Paradela A and B were superficially tilled with a scarifier once a year, and during the time of the experiments they were covered with natural vegetation. V. Figos was a non-tilled olive grove and the soil was mainly covered with stones and natural vegetation. The predominant olive varieties were the autochthonous Cobrançosa, Madural, and Verdeal Transmontana, mainly cultivated for oil production. The trees, approximately 60 years old, were of medium size and were pruned every three years. Their density varied between 7 × 7 m and 10 × 10 m. Thermopluviometric data were obtained from an automatic weather station located 600 to 3000 m from the groves.

### Data collection

The experiments occurred between the beginning of April and the middle of May of 2006 at a periodicity of two or three weeks. Three collections were done at Paradela A and B (1^st and 3rd week of April and 2nd week of May), and two collections at V. Figos (3rd^ week of April and 2nd week of May). The traps used to collect arthropod soil were pitfall traps, measuring 16 cm in height and 9 cm in diameter. 25 traps were used per olive grove randomly distributed in the field in the south side of the canopy at 80 cm from each tree trunk according to Santos et al. (2007). The holes where traps were put were dug carefully with minimal soil and vegetation disturbance, and the top of the trap was at the same level of the soil surface. The traps were used empty without any liquid, and were removed twice a day in order to study two periods: day (07:00 to 19:00) and night (19:00 to 07:00), so as to maximize the numbers of species caught, since some species avoid being active when aggressive species are present (Morris et al. 2002).

The capture rate of a pitfall trap for a species is a function of population density and whether the trap used is appropriate (Melbourne 1999). Therefore, in this work and in equivalent studies (e.g., Lee et al. 2001; Hajek et al. 2007; Gardiner et al. 2010), the trap catches were interpreted as an estimation of the ‘activity density’ of the captured species.

The captured individuals were preserved in 70% ethanol, identified to the taxonomic level of suborder, order, or family, and the total number of each one was recorded. The Formicidae family was identified to species according to Collingwood and Price (1998).

### Data analysis

The number of collected individuals during each of the studied day periods in each grove and sampling period was compared statistically by a Mann-Whitney *U-*test for comparisons between two groups or by a Kruskal-Wallis test for comparisons between three groups. For post-hoc analysis, multiple comparison mean ranks by Fisher's LSD were done, following Maroco (2007). Significance was reported at the level of *p* < 0.05.

Several indices were calculated to provide information on arthropod soil richness and diversity, and are described below:

a) *Richness (S)*

S = total number of taxonomic units collected in the sample;

b) *Shannon index* (H′)


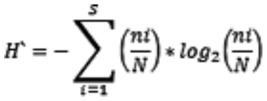


c) *Pielo's evenness index* (E)


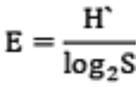


d) *Simpson's index (D)*


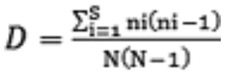


e) *Simpson's index diversity* (1-D)

f) *Morisita index of community similarity* (I_M_)


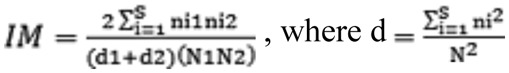


where ni is the number of individuals in the i^th^ taxonomic unit and N is the total number of individuals.

## Results

### Abundance and diversity of soil arthropods

A total of 9725 arthropods, classified into the five classes Chilopoda, Malacostraca, Entognatha, Insecta, and Arachnida, were trapped in the three groves (Table 1). However, only 9654 were considered true soil inhabitants. Insects belonging to the orders Thysanoptera, Homoptera, Diptera, and Hymenoptera (except Formicidae) were excluded in the analysis due to their life behavior.

The order Collembola, within Entognatha, was the most abundant taxa with 70.8% of total captures, and was represented by three suborders: Entomobryomorpha (80.5%), Poduromorpha (14.4%), and Symphypleona (5.1%).

The number of Collembola was significantly different among groves (*p* < 0.01), with a higher abundance in Paradela B than in the other groves (Table 2). Moreover, while no differences were found between sampling dates in V. Figos (*p* = 0.28), the captures of Collembola in the other olive groves were significantly lower in the 1st week of April than in the other two periods (*p* < 0.01, Paradela A; *p* < 0.01, Paradela B) (Table 3).

The class Arachnida represented 20.4% of the total captures. Individuals from this class included those from Acari (84.5%), Opiliones (9.6%), and Araneae (5.9%). In Acari, 42.9% of individuals were Oribatidae.

The abundance of individuals of this class differed significantly among groves for all taxa. Thus, Paradela B had significantly more captures of Acari than Paradela A and V. Figos (*p* < 0.01); V. Figos had a significantly higher number of captures of Opiliones than the other two groves (*p* < 0.01), and Paradela A and Paradela B had significantly more captures of Araneae than V. Figos (*p* < 0.05) (Table 2). The captures of Acari were higher in the 2nd week of May than in the other sampling periods in Paradela B (*p* < 0.01) and V. Figos (*p* < 0.05), while in Paradela A the captures were significantly higher in the last two sampling dates (*p* < 0.01) (Table 3). In all olive groves, Opiliones were only collected in the 2nd week of May (Table 3). The captures of Araneae did not differ significantly between sampling periods in Paradela B (*p* = 0.260) and V. Figos (*p* = 0.926), while in Paradela A captures were significantly higher during the 3rd week of April than in the other sampling dates (*p* < 0.05) (Table 3).

Insecta, with 9.1% of the total captures, was represented by Coleoptera (67.1%), Hymenoptera (Formicidae) (30.1%), Heteroptera (2.4%), Dermaptera (0.4%), and Orthoptera (0.1%). In the Coleoptera order, the families Carabidae (40.7%), Staphylinidae (17.6 %), and Elateridae (16.2%) were separated from the other coleopterans (25.5%). The number of Coleoptera differed significantly among groves (*p* < 0.01), and was significantly higher in Paradela A than in the other two groves. Significant differences were found among groves for Carabidae and Staphylinidae (*p* < 0.01 for both), and was in both cases higher in Paradela A than in the other two groves (Table 2). No significant differences among olive groves were found for Elateridae (*p* = 0.096) (Table 2). The captures of Coleoptera did not differ between sampling periods in V. Figos (*p* = 0.070), while this number was higher in the 2^nd week of May (*p* < 0.05) in Paradela A and in the 3rd^ week of April (*p* < 0.05) in Paradela B (Table 3). Captures of Carabidae did not differ among sampling periods in Paradela A (*p* = 0.528) and V. Figos (*p =* 0.544), while in Paradela B captures were significantly lower in the 3rd week of April (*p* < 0.01) (Table 3). Captures of Staphylinidae did not differ among sampling periods in any grove (*p* = 0.274 for Paradela A; *p* = 0.141 for Paradela B; and *p* = 1.000 for V. Figos) (Table 3). Elateridae were collected only in the 2nd week of May at Paradela A, and no differences were found among sampling periods in Paradela B (*p* = 0.088) or V. Figos (*p* = 0.317) (Table 3).

In Formicidae, a total of 250 individuals were captured belonging to 16 species from 13 genera and three subfamilies (Table 4). Four species appeared in all olive groves, i.e., *Plagiolepis pygmaea* (Latreille 1798), *Crematogaster scutellaris* (Olivier 1792), *Messor bouvieri* (Bondroit 1918), and *Tetramorium semilaeve* (Andre 1883). On the other hand, *Aphaenogaster iberica* (Emery 1908), *Leptothorax angustulus* (Nylander 1856), and *Messor barbarus* (Linnaeus 1767) only appeared in one grove. Dominant species in Paradela A and Paradela B were *T. semilaeve* and *Tapinoma nigerrimum* (Nylander 1856), composing 72.0 and 61.8% of the total Formicidae, respectively. In V. Figos the dominant species were *C. scutellaris* and *T. semilaeve,* which constituted 70.4% of the total Formicidae. They were followed by *M. bouvieri* and *C. scutellaris* in Paradela A and B, and by *Cataglyphis ibericus* (Emery 1906) in V. Figos. The number of captures of Formicidae was significantly different among groves (*p* < 0.05), and was higher in Paradela A than in Paradela B and V. Figos (Table 2). In all olive groves, Formicidae were captured in high numbers in the 2nd week of May, although the difference was only significant in Paradela B (*p* < 0.01) (Table 3). The abundance of the most dominant species of Formicidae did not differ among sampling dates in V. Figos, while abundance in Paradela A and Paradela B was higher in the last sampling for *T. semilaeve* (*p* < 0.01 for Paradela A, *and p* < 0.01 for Paradela B) and *T. nigerrimum* (*p* < 0.01 for Paradela A, and *p* < 0.05 for Paradela B).

From the remaining individuals of Insecta, 20 individuals were from Heteroptera, of which 10 were collected in V. Figos, and five were collected in both Paradela A and Paradela B (Table 1); three from Dermaptera (the European earwig, *Forficula auricularia* Linnaeus) were collected in Paradela A (one) and Paradela B (two) (Table 1); and one individual of Orthoptera (*Gryllotalpa gryllotalpa* Linnaeus) was collected in Paradela A.

Chilopoda and Malacostraca were rare, representing 0.03 and 0.01%, respectively, of the total of arthropods caught, and were captured in V. Figos.

From the collected soil arthropods, a total of 990 (10.3%) were classified as potential predators: Carabidae, Staphylinidae, and Elateridae from Coleoptera, Formicidae from Hymenoptera, as well as Dermaptera, Aranea, Opiliones, and Chilopoda. In each olive grove, the percentage of potential predators was 19.8% in Paradela A, 5.5% in Paradela B, and 19.3% in V. Figos. The Formicidae, Carabidae, Staphylinidae, and Elateridae families were the most abundant potential predators in Paradela A and B, representing 86.5 and 76.4% of the total, respectively. In V. Figos, Opiliones dominated the predatory community with more than 60% of individuals, followed by Formicidae (21.3%). In Paradela B, the total number of arthropods that were potential predators was numerically lower for the second date than the first. This can be related to the rainfall that occurred during the sampling date (about 10.25 mm in 24 hours).

### Abundance and diversity of soil arthropods in relation with the period of the day

Considering all the soil arthropods, the abundance was significantly higher during the day (*p* < 0.01), representing 73.7% of the total captures.

The activity of Collembola was higher during the day (*p* < 0.01) when about 74.0% of individuals were captured. This was also observed in each olive grove, although only significant in Paradela B (*p* < 0.01) and V. Figos (*p* < 0.01) (Table 5). Thus, the percentage of Collembola captured during the day was 56.9% in Paradela A, 78.9% in Paradela B, and 66.6% in V. Figos.

It was also during the day that the activity of Acari (*p* < 0.01), Opiliones (*p* < 0.01), and Araneae (*p* < 0.01) was higher. Thus about 67.7% of Acari, 95.8% of Opiliones, and 62.9% of Araneae were captured in that period. The captures of Acari were higher during the day in Paradela A with 67.1% (*p* < 0.05), in Paradela B with 69.7% (*p* < 0.01), and in V. Figos with 62.9% (*p* < 0.05). Opiliones were captured preferentially during the day, representing 100% in Paradela A (*p* < 0.05), 89.7% in Paradela B (*p* < 0.01) and 96.8% in V. Figos (*p* < 0.01). Araneae captures were higher during the day in Paradela A (*p* < 0.01) and Paradela B (*p* < 0.05), with 65.2 and 69.8% of total individuals collected in this period, respectively, while in V. Figos, no statistical significance was found (*p* = 0.311) for preference between periods (Table 5).

**Figure 1.**
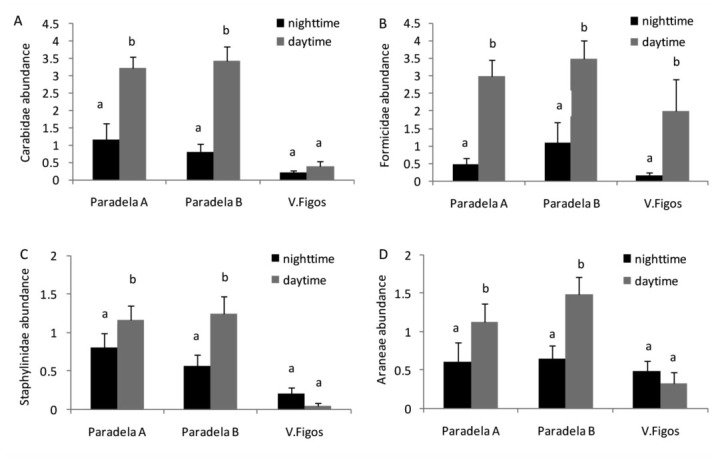
Mean accumulated captures (± SE) during the night and day for (A) Carabidae, (B) Formicidae, (C) Staphylinidae, and (D) Aranea. Histograms sharing the same letter in each olive grove are not significantly different (*p* < 0.05). High quality figures are available online.

Coleoptera also preferred the day (*p* < 0.01 overall, Paradela A, and Paradela B; *p* < 0.05 for V. Figos). The percentage of individuals from Coleoptera collected during the day was 78.2% overall, 78.4% for Paradela A, 77.5% for Paradela B, and 54.6% for V. Figos. About 73.6% of Carabidae in Paradela A (*p* < 0.01) and 81.1% in Paradela B (*p* < 0.01) were captured during the day (Figure 1). The captures of Staphylinidae also were statistically higher during the day in Paradela A (59.2% of captures, *p* < 0.01) and Paradela B (68. 9% of captures, *p* < 0.05) (Table 5).

Hymenoptera (Formicidae) also preferred the day (*p* < 0.01 *overall, p* < 0.01 for Paradela A, *p* < 0.01 for Paradela B, *and p* < 0.01 for V. Figos). The percentage of Formicidae collected during the day was 83.1% overall, 86.2% in Paradela A, 76.3% in Paradela B, and 92.6% in V. Figos (Table 5).

In particular, *T. semilaeve* and *T. nigerrimum* were captured in higher numbers during the day in Paradela A (*p* < 0.01 for *T. semilaeve* and *p* < 0.01 for *T. nigerrimum*) and Paradela B (*p* < 0.01 for *T. semilaeve and p* < 0.05 for *T. nigerrimum*), while in V. Figos the species that was recorded in high number during the day was *T. semilaeve* (*p* < 0.05).

Heteroptera showed a strong preference for the day, i.e., all individuals were collected in this period, while in contrast Dermaptera appeared only at night. The single individual of Orthoptera was recovered during the day. Two of three individuals belonging to Chilopoda were captured at night, as well as the only individual from Malacostraca.

Similar richness was verified between day periods (Table 6). Moreover, while in Paradela A and V. Figos the highest values were recorded during the day, in Paradela B the diversity was higher during the night. However, when making comparisons between the communities of the day and night in each olive grove, the two periods were very similar (> 90%).

## Discussion

This study was designed to obtain information about olive grove arthropod biodiversity. A great number of specimens belonging to different taxa were recovered in all olive groves, though captures were numerically dominated by Collembola. These results were not coincident with other works that found Formicidae as dominant in pitfall traps in olive groves (Santos et al. 2007). In our opinion, this difference is easily explained by the different sampling time. The work conducted by Santos et al. (2007) was performed during the summer. In that season, the olive grove ground is without cover and has very low humidity that favors the disappearance of Collembola and the dominance of other organisms well adapted to such conditions, such as ants. In the present work, with the sampling occurring during the spring period, soil had high moisture due to abundant rainfall, allowing the high densities of collembolans (Shultz et al. 2006). Collembola are considered a biological regulator and have important functions in ecosystems. They are known to feed on bacteria and fungi, mineral soil particles, organic matter, protozoa, and nematodes (Kaneda and Kaneko 2008), and increase soil respiration and accelerate nitrogen mineralization (Kaneda and Kaneko 2008). Collembola are also an alternative prey to generalist predators (Bilde et al. 2000; McNabb et al. 2001; Agustí et al. 2003; Oelbermann et al. 2008) that could enhance predator densities and their impact in biological control (Wise et al. 1999) with particular reference for small spiders (Oelbermann et al. 2008). In this study, the low number of Collembola found in V. Figos is certainly related with the near-complete absence of weeds, high number of stones, and very low amount of organic matter. In fact, the presence of plant material has a great influence in olive soil fauna composition (Castro et al. 1996), and may explain the quantitative poverty of soil entomofauna in V. Figos compared to the other groves where the soil was covered with weeds, some of which were in the flowering period that could provide a nectar and pollen food source and therefore act as a reservoir of alternative prey.

Mites, mainly oribatids, were the second most abundant. Oribatid mites have similar ecological functions as Collembola; they are agents of organic matter decomposition and consequently are important in nutrient recycling. They feed on dead and dying tissues and/or yeasts, bacteria, and algae (Krantz 1978), and are part of the diet of some ant species (Wilson 2005).

The class Insecta represented about 9.0%, composed mainly of coleopterans and ants, while 1.2% of total captures were spiders.

The predatory arthropod community was mainly composed by Carabidae, Staphylinidae, Elateridae, Formicidae, Araneae, and Opiliones, whose numbers varied among olive groves. Centipedes and earwigs were also present, but in low numbers. They are mostly generalist predators, and some of them have been cited as important agents of natural control of insects that spend all or part of their life cycle in the soil, such as the olive fruit fly *B. oleae* (Neuenschwander et al. 1983, 1986; Orsini et al. 2007). The predatory arthropod community was dominated by Carabidae and Formicidae, which is similar to observations by other authors in the same ecosystem (Orsini et al. 2007), although in V. Figos the predatory arthropod community was dominated by Opiliones (more than 60%). Opiliones include in their diet a wide range of arthropods organic matter and fungi (Conrad 2008).

Carabidae represented between 5.9 and 29.6% of total predators captured. Their diet includes a large number of arthropods (Lövei and Sunderland 1996). In olive groves in southern Spain, Morris and Campos (1999) identified *Ditomus capito* s.sp. *haagi* (Heyden) and *Calathus ambiguus* s.sp. *chevrolati* (Gautier), and Neuenschwander et al. (1983, 1986) observed *Carabus banoni* (Dejean.), *Licinus aegyptiacus* (Dejean.), and *Pterostichus creticus* (Frivaldszky) in Greek olive groves.

Other groups with relative importance were Araneae (7.9 to 14.8%) and Staphylinidae (2.4 to 12.9%). Araneae feed almost exclusively on insects (Riechert and Lockley 1984), while most species of Staphylinidae feed on fungi, algae and on decomposing plant matter, whereas others feed on a wide range of many arthropods (Klimaszewski et al. 1996). Morris and Campos (1999) also obtained low captures of spiders and rove beetles. Neuenschwander et al. (1983, 1986) refers *Ocypus olens* O. Muller and *O. fulvipennis* Erichson as the only Staphylinidae out of 12 species that consumed pupae of *B. oleae.*

Dermaptera (*F. auricularia*) and Chilopoda were represented by a low number of individuals. *Forficula auricularia* are omnivores and feed on mosses, lichens, plants, and small living or dead arthropods (Debras et al. 2007). Chilopoda are almost exclusively predatory, feeding on small live arthropods and other invertebrates (Edgecombe and Giribet 2007). Neuenschwander et al. (1983, 1986) observed that some species of Scolopendridae and Lithobiidae predate on *B. oleae* pupae in laboratory experiments.

Elateridae were important in Paradela A (almost 22% from predatory community). The diet of this family is based on plant material (especially roots and tubers) or animals, preying on small soil inhabitant insects (Booth et al. 1990; Farinós et al. 2008) and also pupae of *Anastrepha suspensa* (Hennessey 1997). Due to their number and food preferences they could have importance as predators of olive fruit fly pupae.

The important role of Formicidae is well known in agricultural ecosystems. They participate actively in natural control, pollination, soil improvement, and nutrient cycling. However, the detrimental effect of protecting scales and aphids from their natural enemies is also known (Way and Khoo 1992). In addition, some species also can be considered as ecosystem engineers, since that are responsible for the structure of the soil. Formicidae represented between 21.3 and 31.8% from predator captures. It was constituted mainly of *T. nigerrimum, T. semilaeve,* and *C. scutellaris,* representing 74% of the ants, with some variations between groves. *T. nigerrimum* is one of the most frequent ants in the olive grove of Tras-os-Montes (Northeast of Portugal) (Pereira et al. 2002; Santos et al. 2007) and Granada (South of Spain) (Morris and Campos 1999; Redolfi et al. 1999; Morris et al. 2002).

Agricultural practice is the main influencing factor for the differences observed in olive groves ant communities (Redolfi et al. 1999). Groves with vegetation cover had a great number of ant nests (Redolfi et al. 1999). *Tapinoma nigerrimum* makes shallow ground-nests (Morris et al. 2002), and their absence in V. Figos grove could be due to the high quantity of stones that cover the soil. *Tetramorium semilaeve* is an important and common species in olive groves (Redolfi et al. 1999; Morris et al. 2002; Santos et al. 2007), and was present in all studied groves. *Crematogaster scutellaris,* which was relatively abundant in V. Figos, is also a common species associated with the olive tree. The greatest number of ants occurred in the second week of May, in agreement with other studies (Redolfi et al. 1999; Morris et al. 2002). According to Neuenschwander et al. (1986), many species of ants could attack *B. oleae* larvae as well as pupae inside the fruit and in the soil. Orsini et al. (2007) found that in California, ants were the only predator observed antennating, carrying, or trying to carry olive fruit fly pupae. *Tapinoma nigerrimum* is an omnivorous species that, in addition to eating seeds, also consumes live insects (Redolfi et al. 1999), and thus is characterized as a generalist predator (Cerda and Retana 1988). In the olive grove *Tapinoma nigerrimum* was found to carry live larvae of the olive moth (Morris et al. 1998; Redolfi et al. 1999; Pereira et al. 2002), while *C. scutellaris* was reported as predator of the olive bark beetle, *Phloeotribus scarabaeoides* (Gonzalez and Campos 1990).

The day was found to be clearly preferred by ground predators. This goes against what was found by Morris and Campos (1999), who captured more predators during the night. However, in the case of ants, as in our work, Morris and Campos (1999) and Redolfi (2002) also reported *T. nigerrimum* and *C. scutellaris* as having day activity.

In our opinion, the differences observed are related to the different sampling period and the climatic conditions observed. The work conducted by Morris and Campos (1999) was done in the south of Spain between mid-May and the beginning of September—months with high temperatures during the day that could inhibit the activity of insects. In the present study the average temperature varied between 13.4 ^°^C and 15.6 ^°^C, probably below those recorded by Morris and Campos (1999).

According to several authors, some ground beetles are nocturnal, feeding in the dark and hiding during the day (Lövei and Sunderland 1996). Additionally, most species of rove beetles are nocturnal or avoid the light (Klimaszewski et al. 1996). However, Lövei and Sunderland (1996) explain that this feature can vary with habitat, time of year, temperature, light intensity, and humidity, and the same species may have different behaviors depending on the conditions in which species are inserted.

Considering periodicity, the day was, in general, the period where more arthropods were active; almost 74% individuals were captured in the day period, although species richness (S) was similar. The evenness (E) and diversity indices (H' and 1-D) were higher during the day in Paradela A and V. Figos, while Paradela B was higher at night. Apparently it was conditioned by springtail abundance that occurred in Paradela B during the day. If the indices were calculated without springtails, all indices were higher in day.

In conclusion, these results indicate that during spring in olive groves from Terra Quente, Collembola and Acari made up nearly the entire arthropod soil community. Carabidae and Formicidae were the most abundant predators. Moreover, during this period, the arthropod community was more active during the day.

**Table 1.**
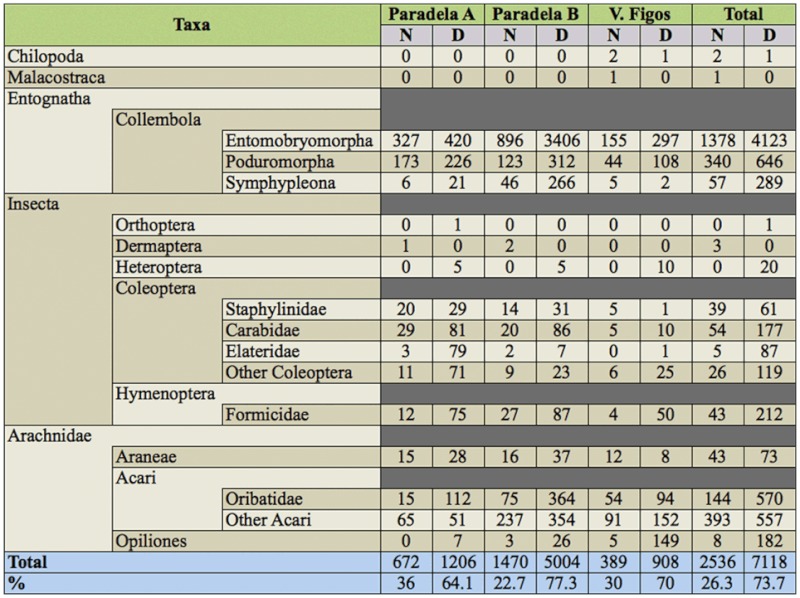
Cumulative number of soil arthropods captured in different day periods (night (N) and day (D)) in the studied olive groves.

**Table 2.**
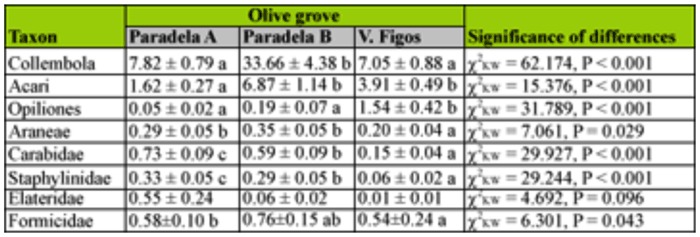
Mean accumulated captures (± SE) for the most abundant taxon collected in pitfall traps in each studied olive grove. Means within the same taxon with different descriptors differ significantly (*p* < 0.05).

**Table 3.**
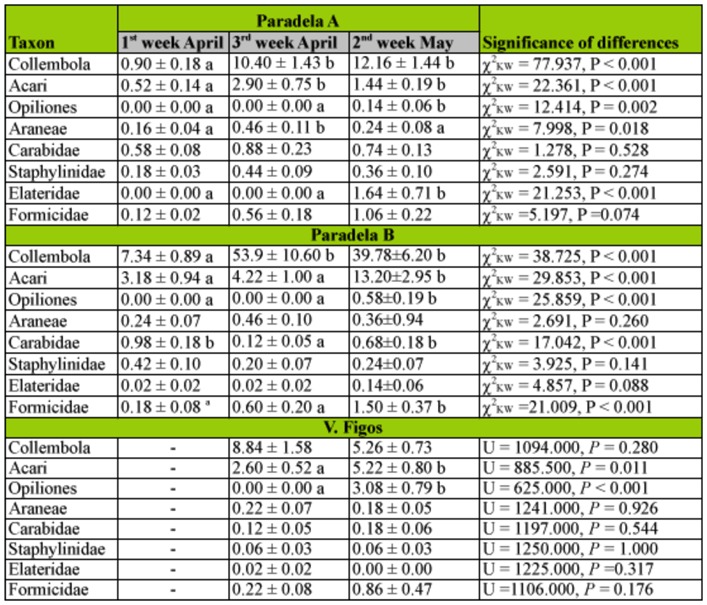
Mean captures (± SE) of the most abundant taxon collected in pitfall traps in the three sampling dates and in each studied olive grove. Means within the same taxon with different descriptors differ significantly (*p* < 0.05).

**Table 4.**
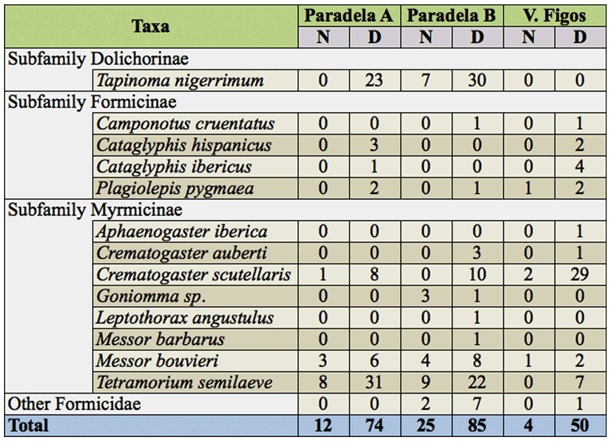
Cumulative number of Formicidae species captured in pitfall traps in the different day periods (night (N) and day (D)).

**Table 5.**
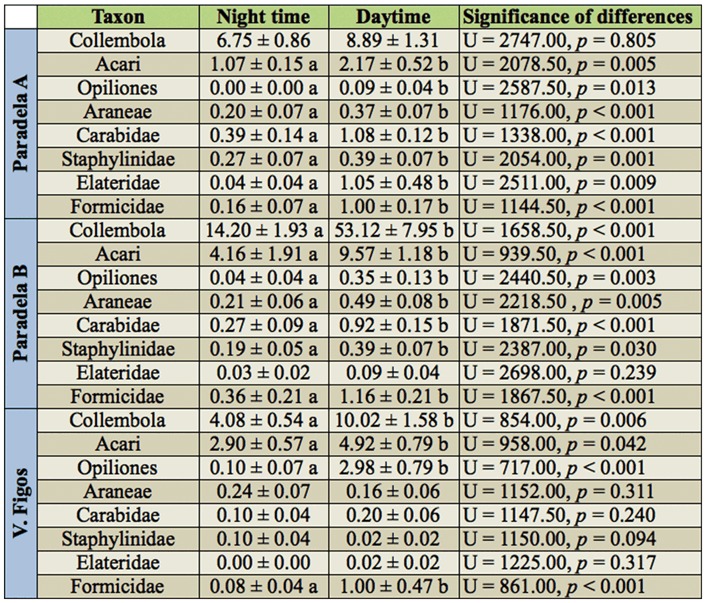
Mean accumulated captures (± SE) during the night and day for the most abundant taxon collected in pitfall traps in each studied olive grove. Means within the same taxon with different descriptors differ significantly (*p* < 0.05).

**Table 6.**
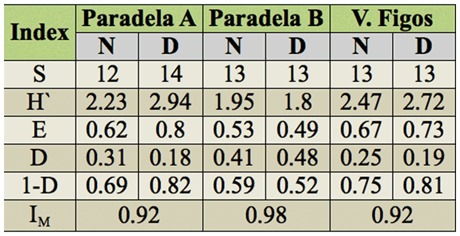
Richness (S), evenness (E), diversity (H', D, and I-D), and community similarity (IM) indices of arthropods soil in the different day periods (night (N) and day (D)) and olive groves in study.
